# Estimating fat content in barred owls (*Strix varia*) with predictive models developed from direct measures of proximate body composition

**DOI:** 10.1093/conphys/coad069

**Published:** 2023-08-30

**Authors:** Ryan C Baumbusch, Katie M Dugger, J David Wiens

**Affiliations:** Oregon Cooperative Fish and Wildlife Research Unit, Department of Fisheries, Wildlife, and Conservation Sciences, Oregon State University, 104 Nash Hall, Corvallis, OR, 97331 USA; U.S. Geological Survey, Oregon Cooperative Fish and Wildlife Research Unit, Department of Fisheries, Wildlife, and Conservation Sciences, 104 Nash Hall, Corvallis, OR, 97331 USA; U.S. Geological Survey, Forest and Rangeland Ecosystem Science Center, 3200 SW Jefferson Way, Corvallis, OR, 97331 USA

**Keywords:** Body condition index, fat score, lipid extraction

## Abstract

Body condition indices and related metrics can help assess habitat quality and other ecological processes, and ideally, these metrics are based on measures of lipids directly extracted from the species of interest. In recent decades, barred owls (*Strix varia*) have become a species of conservation concern as they invaded older forests of the US Pacific Northwest, and caused population declines of the closely related and federally threatened northern spotted owl (*Strix occidentalis caurina*). A simple and effective measure of barred owl body condition could help to understand how habitat quality varies within their new range, which in turn can inform their management and other aspects of their ecology. Using 77 barred owl carcasses collected during experimental removals in Washington and Oregon, USA, we measured the amount of lipid in each specimen with proximate body composition analysis. We then fit and compared (with adjusted *R*^2^ values) alternative linear regression models to estimate the percent lipids in dry mass of the owls based on morphometric body condition indices, a qualitative fat score of subcutaneous breast fat, sex and the time of year females were collected (relative to egg production). Adjusted *R*^2^ values for all models ranged from 0.49 to 0.87, with the best model including mass divided by foot-pad length, fat score, sex and the time of year a female was collected. Most models generated comparable estimates of percent lipids at a population level and we provided correction factors to apply these models when used with live barred owls, allowing for site-specific comparisons of body condition among individuals inhabiting a diversity of environmental conditions.

## Teaser

Using a chemical extraction process, we measured the amount of fat in a sample of barred owl specimens collected in Washington and Oregon, USA. We then developed regression models that estimate the percent of fat in barred owls based on field data.

## Introduction

Energy storage is an important aspect of foraging ecology, where animals try to buffer future energetic demands against spatiotemporal variability in food resources (Brodin and Clark, 2007). Characterizing stored energy through body condition indices can aid conservation and management efforts by informing relationships between individual fitness potential and habitat quality (Johnson, 2007), whereas other measures, such as increased population density, can suggest habitat quality is high when in fact, it is not (Van Horne, 1983; Bock and Jones, 2004; Marra *et al.,* 2015). The importance of body condition has long been recognized in birds (Nice, 1938), especially since larger fat reserves benefit some activities—reproduction and migration (Lindén and Møller, 1989; Lindström and Piersma, 1993)—but hinder others–flight efficiency, foraging ability or predator escape (Hedenström, 1992; Houston and McNamara, 1993; Witter and Cuthill, 1993; Kullberg *et al.,* 1996; Lind *et al.,* 1999). A central challenge in avian body condition research is the ability to accurately quantify energy stores in live birds (Labocha and Hayes, 2012).

Numerous methods index or measure energy stores in birds to assess body condition (McWilliams and Whitman, 2013) while trying to control confounding variables, such as dehydration, recent meals, defecation (Green, 2001) or structural size (Schulte-Hostedde *et al.,* 2005). Ideally, these methods are based on or verified against direct measures of lipid content from carcasses of the species or population under consideration, (e.g. Salewski *et al.,* 2009; Guglielmo *et al.,* 2011). For birds, this means direct measurements of stored lipids are often available for hunted or relatively abundant species (e.g. Labocha and Hayes, 2012), leaving in question the validity of unverified condition indices (Schamber *et al.,* 2009) where lethal sampling may be infeasible or prohibited for rare and endangered species. Birds of prey, for example, have few studies directly measuring lipids (but see Gorney and Yom-Tov, 1994, Massemin and Handrich, 1997, DeLong and Gessaman, 2001). In the Pacific Northwest, USA, large-scale barred owl (*Strix varia*) removal experiments presented an opportunity to directly measure lipids and develop verified body condition indices in a bird of prey, with immediate application for conservation and management.

Following a westward range expansion across North America (Livezey, 2009; Long and Wolfe, 2019), barred owls spread throughout the range of and are outcompeting threatened northern spotted owls (*Strix occidentaliscaurina*; Franklin *et al.,* 2021), prompting consideration of their lethal removal to protect northern spotted owls (USFWS, 2011). Several large-scale experiments assessed the efficacy of barred owl removal and demonstrated conservation value of this action for spotted owls (Diller *et al.,* 2016; Wiens *et al.,* 2021; Hofstadter *et al.,* 2022). Lethally managing barred owls would be a large and controversial undertaking (Lynn, 2018) possibly limited by funding or willingness, necessitating decisions for where and when to conduct removals. Focusing removal efforts towards high-quality barred owl habitat could maximize impact of limited resources to control their population growth rate.

Habitat associations of barred owl territories are well researched (e.g. Hamer *et al.,* 2007; Singleton *et al.,* 2010; Irwin *et al.,* 2018; Jenkins *et al.,* 2019), but few studies have assessed how habitat quality affects their fitness (i.e. survival or reproduction) over the diverse variety of landscapes they use in their expanded range (Wiens *et al.,* 2014; Rossman *et al.,* 2016). While habitat quality is best measured by its effect on fitness, body condition (i.e. the amount of stored fat) can provide a cost- and time-effective alternative index for habitat quality, as condition may correlate with fitness (Johnson, 2007). Past and future barred owl removals provide a ready sample of body condition data to assess how their habitat quality varies. Beyond application to the ‘extremely pressing and complex threat’ (USFWS, 2011) barred owls present to northern spotted owls, a verified body condition index for this wide-ranging owl could answer broader physiological questions related to bird of prey ecology—such as the effect of population density on body condition—and conservation—for example, measuring the effects of environmental contaminants (e.g. rodenticides; Gabriel *et al.,* 2018, Hofstadter *et al.,* 2021) on body condition.

Here, we used barred owl specimens collected as part of a removal study in Washington and Oregon (Wiens *et al.,* 2021) to: 1) directly measure the amount of lipid in a subsample of the owls collected and 2) develop and evaluate linear models that rely on readily obtained information (morphometrics, sex, time of year and fat scores) to estimate the percentage of lipid in individual barred owls. These models provide the ability to compare body condition of barred owls within and among populations that differ in available data, requiring only commonly used tools in ornithological studies (e.g. scales, rulers and calipers).

## Materials and Methods

### Specimen collection

As part of a larger study, we lethally removed 2249 barred owls between 2015 and 2019 from three study areas near Cle Elum, WA, Alsea, OR and Roseburg, OR (Wiens *et al.,* 2021), all of which were long-term northern spotted owl demographic study areas (Franklin *et al.,* 2021). We collected barred owls using 12-gauge shotguns and non-lead ammunition during all times of the year. Collection methods and study areas are described in detail by Wiens *et al.* (2016, 2017). Removal and scientific collection of barred owls was conducted under protocols approved by Oregon State University's Institutional Animal Care and Use Committee and under federal and state Scientific Collection Permits. Upon collection in the field, we measured unflattened wing chord with a ruler to the nearest 1 mm, exposed culmen and foot-pad lengths with calipers to the nearest 0.1 mm, and mass of the whole bird including stomach contents (hereafter ‘field mass’) with a Pesola scale (Pesola, Switzerland) to the nearest 5 g (Bildstein and Bird, 2007). We dissected 1327 owls of the collected owls, where we scored the amount of subcutaneous fat found on the breast, between the ventral feather tracts of each owl using criteria we developed based on observations during dissections (Table 1; hereafter referred to as ‘fat score’). The wing pit has been used to score fat in birds of prey (e.g. DeLong and Gessaman, 2001); however, we observed substantial variation in breast deposits that could be characterized by a small number of categories (Krementz and Pendleton, 1990). We weighed stomach contents of each owl to the nearest 0.01 g and subtracted this from their field mass to obtain ‘carcass mass’.

**Table 1:** Qualitative scoring criteria used to visually characterize the amount of subcutaneous fat on individual barred owls. The area between the 2 ventral feather tracts and the posterior and anterior edges of the rib cage is inspected and scored according to the following criteria. This region should be free of damage (e.g. from gunshot) that would obscure accurate scoring of the fat.

Score	Definition
0	No fat visible under the skin, only muscle.
1	Some fat visible under the skin, but the breast is not entirely covered, and muscle can be seen. This could range from thin, faint deposits of fat flanking the sternum, to thick deposits of fat with one small patch of muscle still exposed.
2	Breast is completely covered with fat, and sternum can be felt through fat by gently placing a finger over the middle of the breast without pressing down.
3	Breast is completely covered with fat, and the sternum cannot be felt through fat by gently placing a finger over the middle of the breast without pressing down.

### Sample selection

Of the owls dissected, 1043 specimens possessed full data (morphometrics, sex and fat score) and were free of severe gunshot damage. We grouped specimens based on sex, fat-score and time of year a female was collected (breeding vs. non-breeding season) and either included all owls that were available in a group at the time of our study or for groups with large numbers of barred owls we randomly selected a maximum of 10 specimens from the group, resulting in a total of 77 barred owls included in the body composition analysis (Table 2). We grouped females into breeding (January–June) and non-breeding seasons (July–December) because body mass and composition can correlate with gonadal hypertrophy and rapid yolk development (Hirons *et al.,* 1984). We found no females with enlarged gonads between July and December. Although testes also enlarge, we did not consider a seasonal effect in males, as testis growth seemed negligible to overall body composition.

**Table 2:** The number of barred owls analysed for body composition within each group of fat score (0–3), sex (male and female) and time of year in relation to breeding cycle for females (BS: Jan-Jun, NBS: Jul-Dec)

Fat Score	Males	Females BS	Females NBS
0	10	4	4
1	10	5	9
2	2	7	10
3	2	5	9

### Aliquot preparation

We plucked all body and flight feathers and trimmed feathers around the ears. Once defeathered, we used poultry shears to cut carcasses, including talons and beaks, into ≤ 2 cm^2^ pieces. Using a 1-hp tabletop meat grinder, we homogenized carcasses with at least two passes through a 4.76-mm die and thoroughly mixed this homogenate by hand. From the homogenate of each owl we took three, approximately 10 g aliquots for body composition analyses.

### Body composition analysis

We adapted standard methods to analyse the proximate body composition of barred owls (Bligh and Dyer, 1959; Dobush *et al.,* 1985; Reynolds and Kunz, 2001). Mass measurements were taken on a Mettler Toledo analytic balance to the nearest 0.0001 g, which we calibrated daily. Aliquots and porous equipment that we weighed were first dried in an oven at 60°C overnight to minimize mass variation from absorbed water in the air due to changes in ambient humidity, with the exceptions that lean aliquots were stored in a desiccation cabinet prior to combustion and weighed directly from the furnace after combustion.

Aliquot wet mass (g) was the mass of the homogenate aliquot prior to drying. We dried each aliquot on an aluminum pan in an oven, until consecutive daily mass measurements were within 0.001 g or the sample mass increased from the previous day indicating that water content in the aliquot was at equilibrium with ambient humidity. This yielded aliquot dry mass (g), and water mass (g) was the difference between wet mass and dry mass. Once aliquots were dry, we ground them with a mortar and pestle, removed and weighed any shot pellets, then loaded the ground dry aliquot into a cellulose extraction thimble and plugged it with a cotton ball rinsed with extraction solvent. To extract lipids from dried aliquots we used a solvent composed of 7:2 hexanes and isopropyl alcohol in a Soxhlet apparatus, running extractions for 22 to 24 hours. This solvent is relatively safer to handle than common alternatives, but it will dissolve both structural (i.e. cellular membranes) and neutral (i.e. stored fat) lipids (Anthony *et al.,* 2000). Lipid mass (g) was the difference in mass of loaded thimbles before and after extraction, and aliquot lean mass (g) was the mass of the loaded thimble after extraction minus the mass of thimble and cotton ball plug measured prior to extraction. We then transferred lean aliquots to ceramic crucibles and combusted them in a muffle furnace for at least 22 hours at 600°C. Bone mass (g) was the mass of the aliquot after combustion, and protein mass (g) was the difference between the bone mass and the lean mass in the crucible prior to combustion. We averaged all mass measurements across the three aliquots for each owl.

### Body condition indices and other covariates

Using the morphometric measurements taken in the field on each specimen (Table 3), we developed four continuous body condition indices (BCIs): 1) carcass mass and carcass mass divided either by 2) wing chord, 3) exposed culmen or 4) foot-pad length. There are several formulations to scale mass by body size (e.g. cube of linear measurement, scaled mass index), but in a post-hoc analysis the BCIs presented here outperformed these formulations. Fat score included four categories, ranked from 0 to 3 (Table 1). Sex was either male or female, and the sex-season parameter had three categories: 1) males, 2) females collected during the egg production (breeding) season from January to June or 3) females collected outside of the egg production (non-breeding) season from July to December. Owls included in this body composition analysis were collected at all times of years, over a 4-year period at three different sites, and while this spatiotemporal variation may influence barred owl condition, it likely would not affect our ability to estimate the lipid content of barred owls (other than the aforementioned differences between breeding and non-breeding season females). Thus, we did not included covariates for year, time of year, or collection site in our models to estimate percent lipid.

**Table 3:** The mean+/−standard deviation and (minimum-maximum) values of all morphometrics used as a body condition index for male and female barred owl for which we directly measured lipid content. Mass was the total carcass mass of each owl after stomach contents were removed.

Metric	Males (n = 24)	Females (n = 53)	Sexes combined (n = 77)
Mass (g)	677+/−65 (575–830)	877+/−109 (652–1170)	815+/−134 (575–1170)
Wing (mm)	315+/−7 (302–331)	324+/−10 (304–347)	321+/−10 (302–347)
Foot-pad (mm)	62+/−2 (58.9–65.5)	65+/−3 (57.7–69.3)	64+/−3 (57.7–69.3)
Culmen (mm)	24+/−1 (22.2–26.5)	26+/−1 (22.8–29.6)	26+/−2 (22.2–29.6)

### Statistical analysis

We used percent lipid in dry mass as our primary response variable in all analyses. While percent lipid in wet mass may provide a more intuitive value relating to the field mass of an owl, uncontrolled variation in water loss between collection and body composition analysis can introduce unaccounted variance in the measurement of wet mass. Thus, percent lipid in dry mass provided a more reliable assessment of body condition in our case. Using the *glm* function in program R (R Core Team 2019), we fit linear models that estimated the percent lipid in dry mass of barred owls. Four univariate models each used one of the BCIs, and additional models used individual BCIs in combination with fat score and/or sex or sex-season as additive coefficients, resulting in a set of 24 models. We compared models with adjusted *R*^2^ to develop predictive models that explained as much variance in our data as possible, rather than simply ranking models with a statistic such as AIC (Burnham and Anderson, 2002).

To evaluate whether models produced different estimates of percent lipid from each other for a given population, we used each model to estimate the percent lipid of all dissected owls with full data (n = 1043). We then performed an ANOVA to compare the estimates of percent lipid across all models and a post hoc pairwise *t*-test with a Bonferroni *P* value correction to compare estimates of percent lipid between models.

**Table 4:** Adjusted *R*^2^ values and parameter coefficients for all generalized linear models fit to estimate the percent lipid in barred owls. Parameters for each model are listed in left hand columns. For models with fat score and/or sex or sex-season, the intercept is for a fat score of 0 and males. For models with sex-season, Female, BS is for females collected during the breeding season (January to June) and Female, NBS is for females collected outside of the breeding season (July to December).

Model Parameters			*R* ^2^	Intercept	BCI	Fat Score			Sex		
				Fat Score 0 Sex Male		1	2	3	Female	Female, BS	Female, NBS
Mass/Foot	Fat Score	Sex-Season	0.87	−34.05	4.64	4.47	13.15	17.08		−11.37	−5.35
Mass	Fat Score	Sex-Season	0.86	−29.52	0.068	4.42	14.02	17.40		−13.47	−7.09
Mass/Wing	Fat Score	Sex-Season	0.86	−27.91	20.61	4.17	14.76	18.68		−11.99	−5.27
Mass/Culmen	Fat Score	Sex-Season	0.83	−20.09	1.28	5.27	17.98	22.38		−9.91	−1.90
Mass/Foot	Fat Score	Sex	0.83	−37.60	4.97	4.57	12.68	16.64	−8.38		
Mass	Fat Score	Sex	0.82	−32.44	0.072	4.58	13.70	17.12	−10.33		
Mass/Wing	Fat Score	Sex	0.82	−30.20	21.65	4.40	14.61	18.67	−8.43		
Mass/Culmen	Fat Score	Sex	0.77	−19.73	1.25	5.92	18.39	23.24	−5.07		
Mass/Foot		Sex-Season	0.76	−60.53	7.50					−12.73	−7.45
Mass		Sex-Season	0.74	−54.44	0.11					−16.05	−10.27
Mass/Wing		Sex-Season	0.70	−52.74	34.48					−13.15	−6.80
Mass/Culmen		Sex-Season	0.56	−42.09	2.29					−8.55	0.18
Mass/Foot		Sex	0.74	−62.79	7.72				−10.08		
Mass		Sex	0.72	−56.50	0.12				−13.17		
Mass/Wing		Sex	0.67	−54.68	35.40				−9.83		
Mass/Culmen		Sex	0.49	−42.37	2.30				−3.34		
Mass/Foot	Fat Score		0.79	−24.39	3.39	5.34	13.43	18.76			
Mass	Fat Score		0.76	−15.85	0.041	5.88	15.14	20.62			
Mass/Wing	Fat Score		0.77	−18.22	14.21	5.37	15.04	20.53			
Mass/Culmen	Fat Score		0.76	−14.46	0.97	6.16	17.66	23.27			
Mass/Foot			0.67	−49.49	6.10						
Mass			0.62	−39.34	0.083						
Mass/Wing			0.61	−42.25	27.71						
Mass/Culmen			0.49	−38.68	2.10						

To provide a correction factor for studies that cannot remove stomach contents, we averaged the stomach content mass of all dissected barred owls and evaluated how well this reduced bias from unmeasured stomach contents. Using our top model, we estimated two new sets of percent lipid for the 77 owls included in the body composition analysis, recalculating BCIs from 1) the field mass (rather than carcass mass) of each owl and 2) the field mass of each owl minus the average stomach content mass. We calculated the bias and mean squared error and tested for differences with paired *t*-tests between the original percent lipid estimates of the top model based on carcass mass and each of the two new sets of estimates.

## Results

The average percent lipid in dry mass across the 77 owls measured was 27% (± 14 SD, geometric mean 23.3%, ± 1.7 SD) and ranged from 8% to 56%. The average standard deviation of the percent lipid of the three samples analysed for each owl was 2.0% (±2.0 SD). The average percent of protein in lean dry mass across all owls was 81% (± 2.0 SD) and ranged from 76% to 86%. The average stomach content mass of the 77 owls included in the analysis of body composition was 13.1 g (±13.6 SD) and ranged from 0.0 to 68.1 g, while the average stomach content mass of all dissected owls was 11.2 g (±12.5 SD, n = 1327 stomachs). The average mass of shot pellets found in aliquots was 0.034 g (± 0.0083 SD), which on average constituted 0.33% of the measured wet mass.

**Figure 1:**
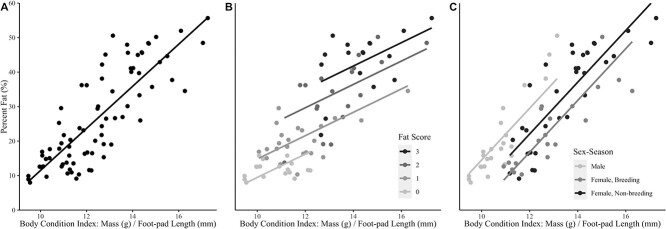
Predicted estimates of the percent lipid in the dry mass of barred owls as a function of the body condition index (BCI) calculated as the mass divided by foot-pad length from 3 linear regression models. Models shown include A) BCI only, B) BCI + fat score and C) BCI + sex season.

All models explained a considerable amount of the variation in the percent lipid of barred owls with adjusted *R*^2^ values ranging from 0.49 to 0.87 (Table 4). Under all parameter combinations (n = 24 models total), models that included mass divided by foot-pad length explained the most variation in percent lipid, and mass divided by culmen explained the least (Table 4; Fig. 1). Including sex in the model almost always improved the amount of variation explained and was further improved by separating females based on the season they were collected (Table 4). Models with fat scores explained more variation than models including only a sex effect (Table 4). The best model (*R*^2^ = 0.87) included a BCI comprised of mass divided by foot-pad length with both fat score and sex-season (Table 4).

The percent lipid estimates for all dissected owls with full data (n = 1043) differed only slightly between each model, with model estimate means for this population ranging from 23.5% to 25.8% (ANOVA; *F* statistic = 7.365, *P* < 0.0001). We excluded all models with culmen length, as they always explained less variation than a model that simply used mass. The post-hoc pairwise t-tests revealed that percent lipid estimates from BCI-only and BCI + fat score models differed statistically from the BCI + sex and BCI + sex-season models (*P* < 0.05 for each pairwise combination) for all but 2 combinations, but these results are likely not biologically meaningful given the small differences in means of model estimates.

Using field mass instead of carcass mass with our top model produced a slight bias (−0.0095, mean squared error = 1.8x10^−4^) and statistical difference (t = −8.5806, *P* < 0.001) in model estimates of percent lipid. While this bias is small and likely negligible, subtracting the average stomach content mass of all dissected owls, 11.17 g, from the field mass reduced bias (−0.0013, mean squared error = 9.49 × 10^−5^) to the point that there was little difference (t = −1.2007, *P* = 0.23) between these model estimates and those using carcass mass.

## Discussion

Our analysis and results demonstrated that a simple body condition index using mass or mass scaled with a basic morphometric measurement can provide accurate estimates of the percent lipid in barred owls. The adjusted *R*^2^ values of the models we fit were comparable to, or better than, similar models fit for other bird species (see review by Labocha and Hayes, 2012). The efficacy of our models in estimating the percent lipid in barred owls was probably facilitated by the large sample of owls collected in a broad range of environmental conditions, which resulted in percent lipid of dry mass that ranged from 8% to 56%. By incorporating easily obtained field information such as the sex or the time of year a female was handled, we improved the accuracy of all models, regardless of the body condition index used. Furthermore, the fat score criteria we developed can substantially improve the accuracy of the BCIs in estimating percent lipid, especially when used in conjunction with sex or time of year (for females). Wing chord is a commonly recorded metric in ornithological studies and routinely used to develop body condition indices (Labocha and Hayes, 2012), but our results suggest that foot-pad length is a better measurement for correcting mass by skeletal size in barred owls. Likewise, some models with mass-only effects explained more variation in percent lipid than mass divided by wing chord.

The upper limit of 56% lipid in dry mass was notably high relative to previous studies in birds, yet percent lipid in wet mass was 30% for this individual, which was more comparable to previous studies (Guglielmo, 2018). Additionally, we selected our solvent for its safer handling properties; however it will dissolve structural lipids (e.g. cell membranes), whereas other common solvents only dissolve neutral lipids (e.g. stored fat; Anthony *et al.,* 2000), resulting in slightly higher measures of lipids. Despite these minor methodological differences, we found relatively high measures of lipids in invasive barred owls compared to other birds of prey (Gorney and Yom-Tov, 1994; Massemin and Handrich, 1997; DeLong and Gessaman, 2001). Indeed, during dissections, we observed owls with substantial fat deposits, such that their abdominal cavities were filled with fat and a 2-cm thick layer of subcutaneous fat covered the breast and abdomen.

In general, female barred owls are larger than males (Mazur and James, 2020), thus we see model coefficients for females were almost always negative compared to males, indicating that for an identical BCI value (especially mass-only) the estimated percent lipid will be higher in males than females. Similarly, coefficients for breeding season females were greater in magnitude (more negative) than the non-breeding season, indicating a change in the proportion of protein to lipid between these seasons, as seen in other birds (e.g. Hohman, 1986). Fat scores explain more variation in percent lipid than just considering sex or sex season, but there was considerable overlap in the percent lipid found across the fat score categories (Fig. 1B), as observed in other bird species (Krementz and Pendleton, 1990; Scott *et al.,* 1994), suggesting that these fat scores are better utilized in conjunction with the BCIs rather than as a standalone measure of body condition.

The data required to estimate percent lipid in barred owls with these models are easily and frequently collected on both live and dead birds, but there are limitations. These models estimate percent lipid in dry mass as a measure of body condition and should not be used to estimate the total fat mass in a whole barred owl. Mass divided by foot-pad length provided the best fitting models, but foot-pad length can be difficult to measure on live birds and impossible on museum study skins with closed feet. Mass divided by wing chord also provided reliable estimates of percent lipid, but wing chord cannot be used if the owl is molting or if the wing was flattened during measurement. Barred owl sex can often be determined through vocalizations (Odom and Mennill, 2010), but if sex is unknown, then we recommend using mass divided by foot-pad length to estimate percent lipid if possible. If stomach content mass cannot be subtracted from field mass, then subtracting the average stomach mass of 11.2 g reduces the very slight bias of unmeasured stomach contents 7-fold. The exact impact of unaccounted for stomach contents varies depending on the model and individual, but, for example, this correction factor (11.2 g) accounts for slightly less than a 1% difference in estimated lipids. Shot pellets constituted 0.33% of the homogenate wet mass, equating to about an extra 3 g in our largest owls collected, less than the precision of our mass measurements (5 g). However, any study applying our models to barred owls not collected with a shotgun may wish to add 0.3 g per 100 g of owl or acknowledge this slight discrepancy.

Many studies of body condition only calculate a size-corrected mass BCI for within study comparison, preventing comparison of body condition across studies using different morphometrics. For instance, a female barred owl with a 335-mm wing chord, 67.1-mm foot-pad and weighing 910 g would yield respective size-corrected mass BCIs of 2.72 and 13.6, affording no comparison. However, our BCI + sex models estimate the percent lipid as 31.8% and 32.1%, respectively. Furthermore, the close agreement across all models in their averaged estimates of percent lipid (23.5–25.8%) for 1043 owls gives us confidence that these models can facilitate comparison of barred owl body condition within and across studies using different morphometric data.

The ability to identify and map habitat quality for barred owls where they have become invasive may be a key component of effective management strategies for this species (Peery *et al.,* 2018). Application of our models to estimate percent lipid in the large number of barred owls collected for the removal studies (Wiens *et al.,* 2021; Hofstadter *et al.,* 2022), as well as owls captured or collected during past and future research, could provide insight into the effect of landscape or forest structural features and population density on individual body condition. Understanding temporal and spatial patterns in barred owl energetics may inform the foraging ecology of this novel, generalist predator and how it interacts within the food web of the Pacific Northwest.
